# Tolerance of Rodents to an Intravenous Bolus Injection of Sodium Nitrate in a High Concentration

**DOI:** 10.3390/biology11050794

**Published:** 2022-05-23

**Authors:** Rachel Katz-Brull

**Affiliations:** 1 Department of Radiology, Hadassah Medical Organization, Faculty of Medicine, Hebrew University of Jerusalem, Jerusalem 9112001, Israel; rkb@hadassah.org.il; 2 The Wohl Institute for Translational Medicine, Hadassah Medical Organization, Jerusalem 9112001, Israel

**Keywords:** sodium nitrate, mouse, rat, intravenous, bolus, dose

## Abstract

**Simple Summary:**

Nitrate is found in many foods and is a common metabolite that is supplied mostly through the diet. Recently, we have found that an analog of this compound, labeled with the stable isotope (non-radioactive) nitrogen-15, is a potentially useful contrast agent for magnetic resonance imaging (MRI), as it does not include a metal component as most other MRI contrast agents. This analog was previously shown with a very high magnetic resonance signal, which is relatively long-lasting, when combined with the new adjunct technology to MRI called hyperpolarization. Prior to serving as a contrast agent for MRI in patients, this agent needs to be tested and validated in small animals. As a prerequisite to such studies, one must ensure that the injection of the naturally abundant agent (not labeled with any isotopes) will be tolerated by the animals. The purpose of the current study was to evaluate the tolerance to an intravenous injection of sodium nitrate in rats and mice, as MRI contrast agents are routinely administered in this way. We have found that a high dose of sodium nitrate can be safely injected into rats and mice. This result opens the way for preclinical MRI studies with sodium nitrate.

**Abstract:**

Nitrate, the inorganic anion NO_3_^−^, is found in many foods and is an endogenous mammalian metabolite, which is supplied mostly through the diet. Although much is known about the safety of sodium nitrate when given *per os*, methodological safety data on intravenous bolus injection of sodium nitrate to rodents are lacking. Recently, we have proposed a new use for nitrate, as a contrast agent for magnetic resonance imaging that will be metal free and leave no traces in the body and the environment further to the imaging examination. It was shown that a stable isotope-labelled analog of this ion (^15^NO_3_^−^), in a sodium nitrate solution form and hyperpolarized state, produces a high magnetic resonance signal with prolonged visibility. Therefore, sodium nitrate was targeted for further preclinical development in this context. In the absence of methodological safety data on the potential effects of a high concentration sodium nitrate bolus intravenous injection into rodents, we carried out such an investigation in mice and rats (*n* = 12 of each, 6 males and 6 females in each group, altogether 24 animals). We show here that an intravenous bolus administration of sodium nitrate at a concentration of 150 mM and a dose of 51 mg/Kg does not lead to adverse effects in mice and rats. This is the first investigation of the tolerance of rodents to an intravenous injection of sodium nitrate.

## 1. Introduction

Nitrate, the inorganic anion NO_3_^−^, is found in many foods and mostly in vegetables [1]. Nitrate is an endogenous mammalian metabolite, which is supplied mostly through the diet [2,3,4]. In the body, nitrate is reduced to nitrite [5,6], mainly by the bacterial nitrate-reductase enzymes present in saliva [2,5,6,7,8,9,10]. In mammalian tissues, the rate of this reduction is low [9]. The produced nitrite can then be reduced to the vasodilator nitric oxide (NO) [2,3,5].

Recently, we have proposed a new use for nitrate, as a contrast agent for magnetic resonance imaging (MRI) that will be metal free and leave no traces in the body and the environment further to the imaging examination [11]. This is in contrast to the currently used MRI contrast agents that are Gadolinium based and show deposits in the body [12,13,14,15] and accumulation in the environment [16]. The global annual number of contrast-enhanced MRI examinations—about 30 million [17]—justifies developing new MRI agents that do not pose these hazards.

In order to serve as such an MRI contrast agent, a stable isotope-labelled analog of this ion (^15^NO_3_^−^), in a sodium nitrate solution form, was investigated in hyperpolarized magnetic resonance conditions and showed a high magnetic resonance (MR) signal and prolonged visibility, potentially useful for clinical examinations [11]. Hyperpolarized tracers show a very high meta-stable signal on MRI for tens of seconds to minutes. For this reason, such imaging agents must be administered in a bolus intravenous injection (as done routinely on contrast-enhanced MRI). As in the case of other MRI contrast agents, the nitrate-based agent would require preclinical validation. The hyperpolarized MR signal is linearly related to the polarization level of the agent [18] and to its amount. Due to the latter, it is desirable to inject the contrast agent in the highest concentration that is safe for injection.

The current study was carried out towards such a preclinical validation. Although much is known about the safety of sodium nitrate when given *per os* [19,20], we could not find methodological safety data on intravenous sodium nitrate bolus injection to rodents in the literature. Therefore, we set to investigate this. We note that we did not expect to find severe adverse effects of such an injection as data obtained from a study in humans provided a favorable starting point. In a study by Ellen et al. [21], twelve volunteers received 750 mL of an aqueous solution containing 9.5 g of NaNO_3_, intravenously, in about 60 min. None of the persons who received NaNO_3_ showed any side-effects after treatment. The concentration of the above solution is 149 mM, which is isosmotic and therefore suitable, from an osmotic perspective, for injections to humans/mammals. We studied here the acute possible effects of a bolus intravenous injection of a similar solution in rats and mice.

In principle, toxicity from a nitrate injection is expected to be acute only. This is because the potential toxicity of nitrate is likely predominantly related to its reduction to nitrite and not to nitrate itself. Nitrite (NO_2_^−^) is a bioactive ion in mammals and serves as a reservoir of nitric oxide (NO). On the other hand, nitrate is relatively inactive biologically [9], and as such was used as a control in studies of nitrite activity [22]. Importantly, in a 2-year study in rats, both sodium nitrate and sodium nitrite did not exert carcinogenic effects [23]. The half-life of nitrate in the circulation is about 5–6 h while that of nitrite is 20 min [2]. In agreement with the latter, potential adverse effects were studied here for 1.5 h following injection (as opposed to days or more). In addition, as intravenously administered sodium nitrate will be first distributed in the blood, we note that about 60–70% of the plasma nitrate is excreted in the urine [1,24] and only a few percent of circulating nitrate is converted into nitrite [1].

## 2. Materials and Methods

### 2.1. Materials

Sodium nitrate analytical standard, ≥99.995% purity based on trace metals analysis, was purchased from Sigma-Aldrich (Rehovot, Israel). Double-distilled water (DDW) were supplied by Hadassah Medical Center’s central pharmaceutical facility. Isoflurane was obtained from the Institutional Authority for Biological and Biomedical Models of the Hebrew University (Jerusalem, Israel). Medical-grade saline was purchased from B. Braun (Melsungen, Germany).

### 2.2. Solution for Intravenous Injection

An isosmotic solution of sodium nitrate solution (150 mM) was prepared by combining 487.2 mg of sodium nitrate with 38.2 mL of DDW. The solution was sterilized by passing the entire volume through a 0.45 µM filter (Minisart^®^ Syringe Filter, Sartorius, Germany), placed in sterile tubes under sterile conditions in a sterilized biological hood, and kept refrigerated (<8 °C up to 7 days), prior to injection into the animals. On each experimental day, 3 animals were injected with a solution that was stored in one tube and the remaining solution was discarded. A total of 1 mL of this solution was injected per rat, in a dose of 51 mg/Kg (calculated based on an average 250 g weight for the rats). A hundred μL of this solution was injected per mouse, resulting in the same dose of sodium nitrate (51 mg/Kg), considering an average 25 g weight for the mice.

As a control for the sodium nitrate injection, weight- and sex-matched animals were injected with the same volume of medical-grade sterile saline (1 mL for rats and 100 μL for mice).

### 2.3. Animals

Female Sprague-Dawley rats (*n* = 12, 6 males and 6 females) and Institute of Cancer Research (ICR) mice (*n* = 12, 6 males and 6 females) were obtained from the Hebrew University Authority of Biological and Biomedical Models. The joint ethics committee (IACUC) of the Hebrew University and Hadassah Medical Center approved the study protocol for animal welfare (Protocol Number MD-19-16068-3). The study was originally planned as a dose–response study; however, further to favorable data found later in the literature [21], and in order to save animal lives, we have decided to test the highest possible isosmotic concentration of sodium nitrate (150 mM) without prior investigation of lower doses. The Hebrew University is an AAALAC International accredited institute. Care was taken to minimize pain and discomfort to the animals. Animals were housed in the animal facility 3–5 days after delivery for acclimatization and fed *ad libitum*.

### 2.4. Experimental Workflow

**Figure 1:**
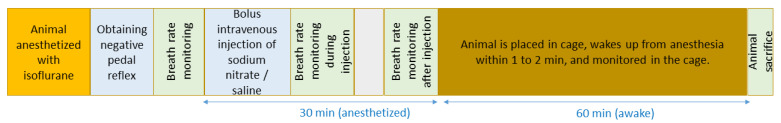
A scheme of the experimental workflow.

The animal was anesthetized with isoflurane using a gas anesthesia system (Somnosuite, Kent Scientific, Torrington, CT, USA). Upon obtaining a negative pedal pain reflex, a tail vein injection of sodium nitrate or saline (see *Solution for intravenous injection* above) was performed. In the tail vein injections, 25 G needles were used for rats, and 27 G needles were used for mice. Thirty minutes after the injection, the animal was taken out of anesthesia, placed in an individual cage, and woke up. The animals were monitored visually in the cage for 1 h (overall, until 1.5 h after the injection). Breath rate was monitored visually before, during, and after the injection, while the animal was anesthetized. At the end of the experiment, the animals were sacrificed with a CO_2_ overdose. This workflow is described in Figure 1.

### 2.5. Statistical Analysis

Statistical analysis was performed with Excel (Microsoft, Ra’anana, Israel). Comparison between conditions were done using a two-tailed, paired Student’s *t*-test.

## 3. Results

While anesthetized, the breath rate of the mice was not altered during the sodium nitrate intravenous bolus injection or afterwards (Table 1). For male rats, the breath rate was reduced during the injection and afterwards. For female rats, the breath rate was reduced after the injection compared to the breath rate prior to the injection (Table 1). Changes in breath rate were not observed in rats or mice during or after saline injection (Table 2).

Animals were inspected immediately at the end of anesthesia (upon awakening)—after half an hour from the injection, and then for 1 h more (altogether 1.5 h monitoring of each animal further to the injection of sodium nitrate or saline).

All 24 animals woke up from anesthesia normally and did not show any clinical or behavioral signs during 1 h after awakening from anesthesia. Their body position was normal, their eyes were open, their walking in the cage was normal, their back position was straight (not convex), their fur was normal (not standing on end), and there were no signs of over-licking or any other unusual signs.

## 4. Discussion

This study shows that an intravenous bolus administration of sodium nitrate at a concentration of 150 mM and a dose of 51 mg/Kg is well tolerated by rats and mice, and does not lead to adverse effects. The reduction in breath rate observed in rats was indeed significant, but not crucial. In addition, due to the small number of animals, we hesitate to draw conclusions from this finding. At any rate, this temporary change did not appear to affect the behavior of the awake animals and this suggests that indeed there were no adverse effects to the injection. For this reason, we did not find it ethically warranted to investigate this in more rats. This result suggests that other isosmotic solutions of sodium nitrate with a lower sodium nitrate dose will not lead to adverse effects either. In conclusion, this study supports further preclinical research into sodium nitrate as a novel MRI contrast agent in anesthetized rodents.

This result may also be useful for other studies of sodium nitrate as its potential beneficial effects gained interests since the discovery that nitrate reductase activity occurs also in mammalian tissues and not only in bacteria [9,25]. Benefits of sodium nitrate were also reported in the context of several diseases and conditions such as diabetes [26] and heart failure [27].

Study limitations: This study was limited in scope because its aim was very focused—to test whether sodium nitrate could be injected as a bolus of a high concentration to mice and rats without adverse effects. This was done as a first step prior to MRI investigations in mice and rats using this agent when labeled with nitrogen-15 at a hyperpolarized state, as previously suggested [11]. The conclusions that can be drawn from this study regarding the safety of such an injection are limited due to short- and long-term-related factors. With regard to the short-term factors, breath rate was monitored visually by a single observer and not electronically; heart beats, oxygen saturation, and blood pressure were not monitored. With regard to the long-term factors, distribution in body organs and removal from the body were not assayed. In addition, prior to clinical MRI studies, this study should be repeated in a larger number of animals, in larger animals, in awake animals, and in humans, using the array of biochemical and bio-distribution assays required for regulatory approval for human use.

## 5. Conclusions

In conclusion, this is the first investigation of the tolerance of rodents to an intravenous injection of sodium nitrate.We conclude that a bolus injection of sodium nitrate at a high, yet isosmotic, concentration is well tolerated by mice and rats.

## Figures and Tables

**Table 1:** Averaged data—animal weight and breath rate before, during, and following a sodium nitrate injection *, under anesthesia.

Animal Group	Rat/ Mouse	Male/ Female	Weight (g) **	Breath Rate before Injection	Breath Rate during Injection	Breath Rate Following Injection
1	Rat	M	247 (5)	81 (5)	63 (9) ^a^	60 (7) ^b^
2	Rat	F	239 (9)	85 (10)	77 (15)	51 (6) ^c^
3	Mouse	M	25	105 (34)	95 (10)	88 (16)
4	Mouse	F	25	140 (17)	128 (29)	105 (28)

Values are presented as the mean (± standard deviation). Each group consists of 3 animals. Individual data are provided in Table S1 (Supplementary Information). * The sodium nitrate solution (150 mM) was injected intravenously as a bolus as described in the Materials and Methods. ** The weight of the mice is reported as per their order. The mice were inadvertently not weighed individually. Typically, when ordered at this weight, mice weigh 25 ± 1 g. ^a^, *p* = 0.020, comparing breath rate before and during injection. ^b^, *p* = 0.004, comparing breath rate before and after injection. ^c^, *p* = 0.006, comparing breath rate before and after injection. All statistical tests were done with a two-tailed, paired Student’s *t*-test.

**Table 2:** Averaged data—animal weight and breath rate before, during, and following a saline injection *, under anesthesia.

Animal Group	Rat/ Mouse	Male/ Female	Weight (g)	Breath Rate before Injection	Breath Rate during Injection	Breath Rate Following Injection
1	Rat	M	246 (3)	87 (7)	81 (6)	77 (8)
2	Rat	F	244 (3)	52 (8)	47 (2)	47 (10)
3	Mouse	M	24 (1)	119 (35)	120 (4)	107 (30)
4	Mouse	F	22 (1)	117 (34)	116 (17)	111 (27)

Values are presented as the mean (± standard deviation). Each group consists of 3 animals. Individual data are provided in Table S2 (Supplementary Information). * Medical-grade saline was injected intravenously as a bolus as described in the Materials and Methods.

## Data Availability

No new data were created or analyzed in this study. Data sharing is not applicable to this article.

## Supplementary Materials

The following supporting information can be downloaded at: https://www.mdpi.com/article/10.3390/biology11050794/s1, Table S1. Individual animal breath rate before, during, and following a sodium nitrate injection, under anesthesia; Table S2. Individual animal breath rate before, during, and following a saline injection, under anesthesia.
