# Supplementary material for: Tolerance of Rodents to an Intravenous Bolus Injection of Sodium Nitrate in a High Concentration
Source: Biology (Basel). 2022 May 23;11(5):794. doi: 10.3390/biology11050794 (PMC9138515; doi:10.3390/biology11050794)
Supplement: Supplementary file 1 [file biology-11-00794-s001.zip › biology-1636944-supplementary.pdf]

Supplementary information

Table S1. Individual animal breath rate before, during, and following a sodium nitrate injection*, during anesthesia.

Animal number	Rat/ Mouse	Male/ Female	Weight (g)**	Breath rate before Injection	Breath rate during Injection	Breath rate following Injection	Sodium Nitrate Dose (mg/kg)
1	Rat	M	252	84	68	64	50.6
2	Rat	M	243	84	68	64	52.5
3	Rat	M	245	76	52	52	52.0
4	Rat	F	248	84	88	52	51.4
5	Rat	F	230	76	84	44	55.4
6	Rat	F	240	96	60	56	53.1
1	Mouse	M	25	84	104	104	51.0
2	Mouse	M	25	144	84	72	51.0
3	Mouse	M	25	88	96	88	51.0
4	Mouse	F	25	148	96	136	51.0
5	Mouse	F	25	152	136	80	51.0
6	Mouse	F	25	120	152	100	51.0

* The sodium nitrate solution (150 mM) was injected intravenously as a bolus as described in the Materials and Methods.

** The weight of the mice is reported as per their order. The mice were not weighed individually (inadvertently). Typically, when ordered at this weight, mice weigh 25 ± 1 g.

Table S2. Individual animal breath rate before, during, and following a saline injection*, during anesthesia.

Animal number	Rat/ Mouse	Male/ Female	Weight (g)**	Breath rate before Injection	Breath rate during Injection	Breath rate following Injection
1	Rat	M	244	80	76	80
2	Rat	M	250	94	80	84
3	Rat	M	244	88	88	68
4	Rat	F	245	44	48	56
5	Rat	F	247	60	48	36
6	Rat	F	241	52	44	48
1	Mouse	M	23.5	148	120	72
2	Mouse	M	25.3	128	124	128
3	Mouse	M	23.4	80	116	120
4	Mouse	F	20.9	148	96	88
5	Mouse	F	21.2	80	128	104
6	Mouse	F	22.4	124	124	140

* Medical grade saline was injected intravenously as a bolus as described in the Materials and Methods.
